# Bactrian camels shed large quantities of Middle East respiratory syndrome coronavirus (MERS-CoV) after experimental infection^*^

**DOI:** 10.1080/22221751.2019.1618687

**Published:** 2019-05-23

**Authors:** Danielle R. Adney, Michael Letko, Izabela K. Ragan, Dana Scott, Neeltje van Doremalen, Richard A. Bowen, Vincent J. Munster

**Affiliations:** a Department of Biomedical Sciences, Colorado State University, Fort Collins, CO, USA; b Rocky Mountain Laboratories, National Institute of Allergy and Infectious Diseases, National Institutes of Health, Hamilton, MT, USA

**Keywords:** MERS-CoV, Bactrian camel, dromedary camel, virus shedding, natural reservoir

## Abstract

In 2012, Middle East respiratory syndrome coronavirus (MERS-CoV) emerged. To date, more than 2300 cases have been reported, with an approximate case fatality rate of 35%. Epidemiological investigations identified dromedary camels as the source of MERS-CoV zoonotic transmission and evidence of MERS-CoV circulation has been observed throughout the original range of distribution. Other new-world camelids, alpacas and llamas, are also susceptible to MERS-CoV infection. Currently, it is unknown whether Bactrian camels are susceptible to infection. The distribution of Bactrian camels overlaps partly with that of the dromedary camel in west and central Asia. The receptor for MERS-CoV, DPP4, of the Bactrian camel was 98.3% identical to the dromedary camel DPP4, and 100% identical for the 14 residues which interact with the MERS-CoV spike receptor. Upon intranasal inoculation with 107 plaque-forming units of MERS-CoV, animals developed a transient, primarily upper respiratory tract infection. Clinical signs of the MERS-CoV infection were benign, but shedding of large quantities of MERS-CoV from the URT was observed. These data are similar to infections reported with dromedary camel infections and indicate that Bactrians are susceptible to MERS-CoV and given their overlapping range are at risk of introduction and establishment of MERS-CoV within the Bactrian camel populations.

## Introduction

Middle East respiratory syndrome coronavirus (MERS-CoV) circulates in dromedary camels and is responsible for severe respiratory disease in humans [1–9]. Experimental infections and field observations indicate that infected dromedaries shed large quantities of virus via nasal secretions and that signs of clinical disease are limited to rhinorrhea and a mild elevation in body temperature [10–12]. Infectious virus was detected in nasal swabs collected during the first week from experimentally infected dromedaries and RNA was detected for 35 days post-infection (dpi) [10,11,13,14]. In addition, productive infections after experimental inoculation with MERS-CoV of new world camelids, llama's and alpaca, have been reported suggesting a broad host tropism of MERS-CoV for camelids [13,15,16].

**Figure 1.**
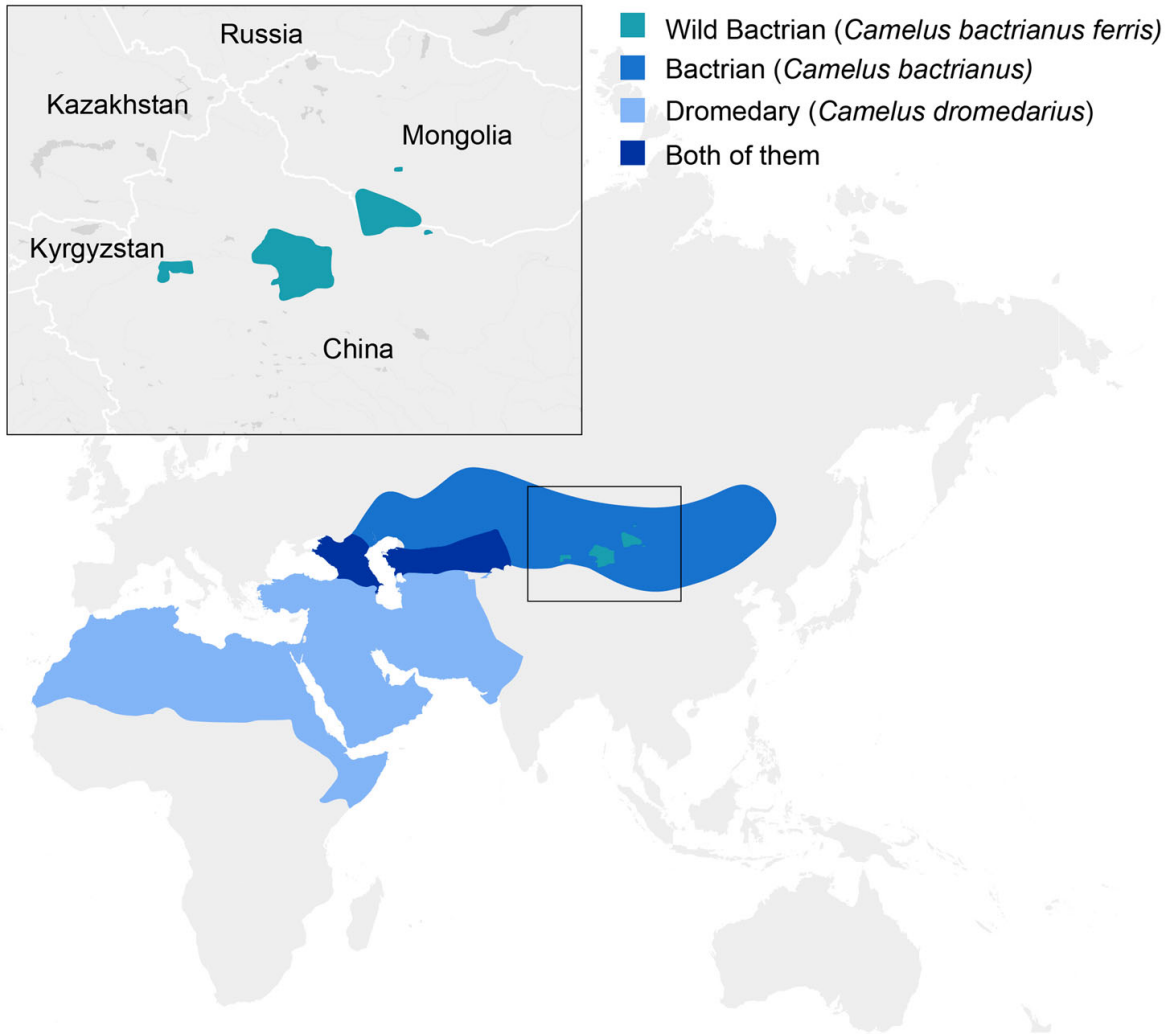
Geographical distribution of Bactrian camels. Geographical distribution of dromedary camels, Bactrian camels and wild Bactrian camels, including the area in which dromedary camels and Bactrian camels are co-localized.

Old world camelids include dromedary and Bactrian camels. Bactrian camels consist of two subspecies: the critically endangered wild Bactrian camels (*Camelus bactrianus ferus*), and domesticated Bactrian camels (*Camelus bactrianus bactrianus*). They are distributed over Western, Central and South Asia, including an area that overlaps with the distribution of dromedary camels (Figure 1A) [17,18]. A 2014 survey of 30 Bactrian camels from 12 herds in southern Mongolia failed to detect circulation of MERS-CoV either by qRT-PCR on nasal swabs or serology, indicating either that the virus was not circulating in these herds or that these animals are not susceptible to infection [19]. Similarly, a 2015 survey of 190 Bactrian camels from 10 herds in the West Inner Mongolia Autonomous Region of China failed to detect circulation of MERS-CoV by qRT-PCR or serology [20]. Sero-surveillance conducted in Kazakhstan did not detected any evidence for circulation of MERS-CoV in 455 sampled dromedary camels and 95 Bactrian camels [21]. In order to determine the susceptibility of Bactrian camels to infection with MERS-CoV and the potential risk for introduction of MERS-CoV in areas with Bactrian camels, we inoculated two Bactrian camels and monitored them for nasal shedding and seroconversion and compared the response to experimental historic data from dromedary camels.

## Materials and methods

*Ethics Statement:* All experiments were approved by the Colorado State University Institutional Animal Care and Use Committee. Work with infectious MERS-CoV strains under ABSL3 conditions was approved by the Institutional Biosafety Committee (IBC). Inactivation and removal of samples form high containment was preformed according to IBC-approved protocols.

### Study design

Two intact adult male domesticated Bactrian camels were obtained through private sale and housed in an Animal Biosafety Level 3 facility for the entirety of the experiment. They had access to food and water ad libitum and were observed at least once daily. Each camel was sedated with xylazine and inoculated intranasally with MERS-CoV (strain HCoV-EMC/2012) diluted in phosphate-buffered saline for a total dose of 10^7^ plaque-forming units (PFU) delivered in 3 mL per nare. Nasal swabs were collected from both camels daily 1–5 days post inoculation (dpi), and from Bactrian camel 1 on day 7, 14, 21 and 28. Serum samples were collected from both animals on day 0, and from Bactrian camel 1 on 7, 14, 21, and 28 dpi. Bactrian camel 2 was euthanized on 5 dpi and Bactrian camel 1 on 28 dpi; Bactrian camel 2 was necropsied and tissues processed for virus titration and histopathology.

### RNA extraction and quantitative PCR

RNA was extracted from swabs, fecal samples and serum samples using the QiaAmp Viral RNA kit (Qiagen) according to the manufacturer's instructions. For detection of viral RNA, 5 μl of RNA was used in a one-step real-time RT–PCR upE assay using the Rotor-GeneTM probe kit (Qiagen) according to manufacturer's instructions [22]. Standard dilutions of a titered virus stock were run in parallel, to calculate TCID_50_ equivalents in the samples.

### Virus titration and serology

Shedding and tissue distribution of MERS-CoV was evaluated by virus titration on Vero cells as described previously [10,13]. In short, swab samples in viral transport medium and homogenized tissues (∼10% w/v) were titrated for MERS-CoV virus by plaque assay. Briefly, ten-fold serial dilutions of samples were prepared in BA-1 medium containing 100 mg gentamicin, 200,000 U penicillin G, 100 mg streptomycin and 5 mg amphotericin/L, and 0.1 ml volumes were inoculated onto confluent monolayers of VeroE6 cells grown in six-well cell culture plates in duplicate. The inoculated cells were incubated for 45 min at 37°C in 5% CO_2_ in air and then overlaid with 2 ml/well of MEM without phenol red and containing 0.5% agarose, 2% fetal bovine serum and antibiotics as described above. Two days after the initial overlay, a second overlay was added which was identical to the first except for inclusion of neutral red (33 mg/L). Plaques were counted on days 1 and 3 after the second overlay and virus titers expressed as plaque-forming units (pfu) per ml.

### Plaque reduction neutralization assay

Neutralizing antibodies were detected using a plaque reduction neutralization assay (PRNT) using 90% plaque reduction as previously described [10,13]. In short, camel sera were heat inactivated for 30 min at 56°C and two-fold dilutions beginning at 1:5 prepared in BA-1 medium. These samples were mixed with an equal volume of MERS-CoV to obtain a virus concentration of 100 pfu/0.1 ml and an initial serum dilution of 1:10. The virus-serum mixtures were incubated at 37°C for 60 min, then inoculated onto VeroE6 cells as described for plaque assay. Plaque reduction neutralization assay (PRNT) titers were calculated as the reciprocal of the highest dilution that resulted in >90% neutralization of virus relative to no serum control samples.

### Histopathology and immunohistochemistry

Tissues were collected from Bactrian camel 2 and fixed for >7 days in 10% neutral-buffered formalin and embedded in paraffin using standard techniques. Histopathologic evaluation was performed on slides stained with hematoxylin and eosin. Other slides were deparaffinized and immunostained using a rabbit polyclonal antiserum to MERS-CoV (EMC/2012) as primary antibody. Slides were evaluated by a board-certified veterinary pathologist.

## Results

### Dipeptidyl peptidase 4 (DPP4) analysis

**Figure 2.**
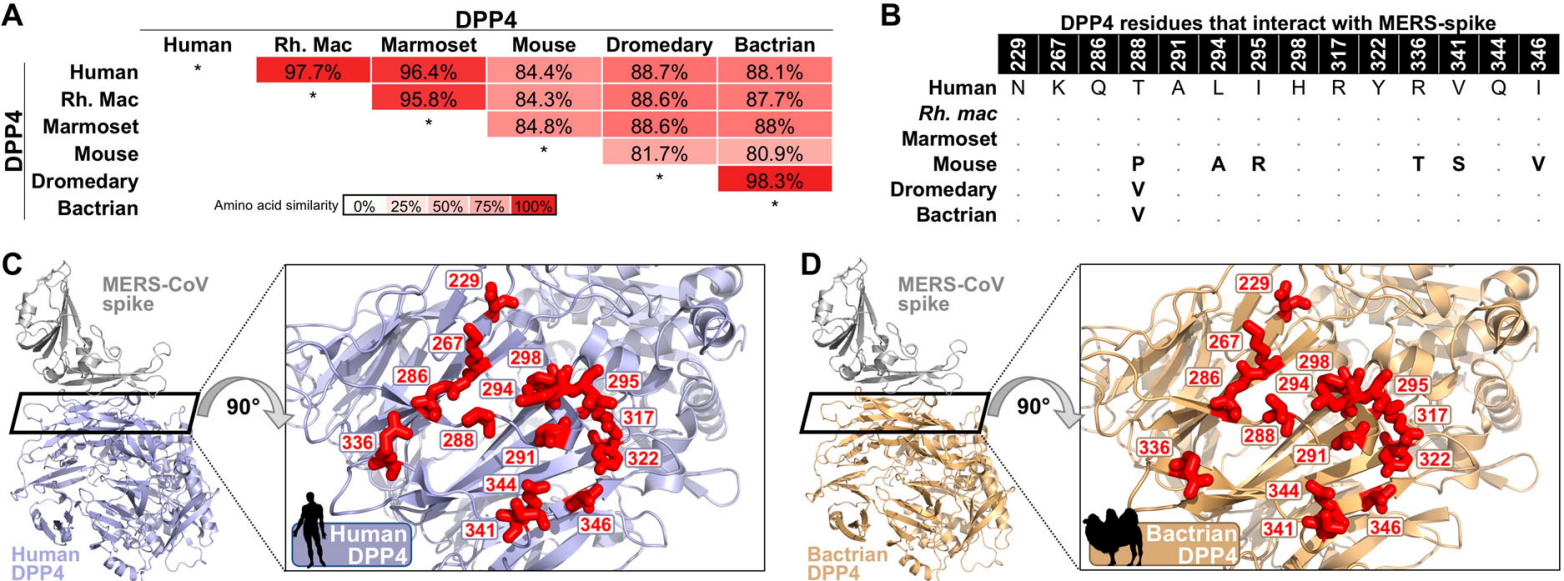
Bactrian camel dipeptidyl peptidase 4 (DPP4) analyses. (A) DPP4 amino acid sequence identity matrix. White indicates no similarity, dark red indicates 100% similarity. (B) DPP4 species amino acid variation within the 14 described contact points with MERS-CoV spike. (C) Co-structure of MERS-CoV spike interaction with human DPP4 (PDB: 4L72). Inset shows a top-down view of the 14 spike-contact points on DPP4 (coloured in red). (D) Co-structure of MERS-CoV spike interaction with the predicted structure of Bactrian camel DPP4 (Swissmodel). Inset shows a top-down view of the 14 spike-contact points on DPP4 (coloured in red).

The host receptor of MERS-CoV, DPP4, was compared between Bactrian camels and species known to be susceptible (human, non-human primates and dromedary camels) and not susceptible (mice) [23]. Bactrian DPP4 was 98.3% identical to the dromedary camel DPP4 on the amino acid level, and only varied from human DPP4 by one amino acid in the residues that specifically interact with the MERS-CoV spike (Figure 1B and C). A modelled co-structure of MERS-CoV spike and Bactrian camel DPP4 showed that the interfacing amino acids were oriented similarly to the known co-crystal structure of MERS-CoV spike and human DPP4. The 100% identity of the 14 amino acid residues between dromedary camel and Bactrian camel DPP4 in the interaction regions indicates that MERS-CoV RBD should bind to Bactrian camel DPP4. This suggests that the MERS-CoV spike would be able to utilize Bactrian camel DPP4 (Figure 2D and E).

### Clinical disease

**Figure 3.**
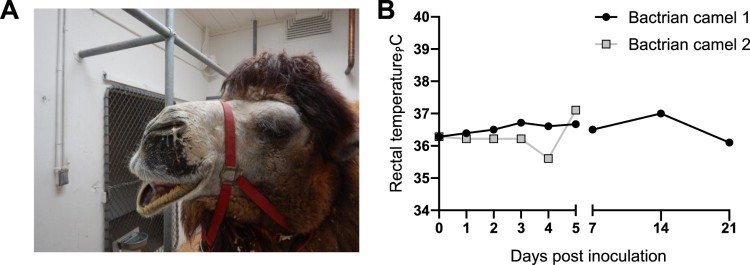
Clinical signs in Bactrian camels inoculated with Middle East respiratory syndrome coronavirus (MERS-CoV). (A) Nasal discharge observed in Bactrian camel 2; both Bactrian camels displayed nasal discharge during the experiment (B) Rectal temperature during the experiment. Rectal temperatures are indicated for each camel by lines with geometric shapes.

Two mature Bactrian camels were inoculated with 10^7^ TCID_50_ of a human isolate of MERS CoV (strain HCoV-EMC/2012) [1] via the intranasal route. Bactrian camel 1 developed noticeable bilateral discharge beginning on 5 days dpi that continued through 7 dpi. Nasal discharge was observed in Bactrian camel 2 on 3 dpi after the animal became agitated due to handling; coughing and bilateral discharge was observed on 4 dpi, and the animal had bilateral discharge at necropsy on 5 dpi (Figure 3A). Body temperature for both animals remained within normal limits following virus inoculation and for the duration of the study (Figure 3B).

### Viral shedding and tissue distribution of MERS-CoV

**Figure 4.**
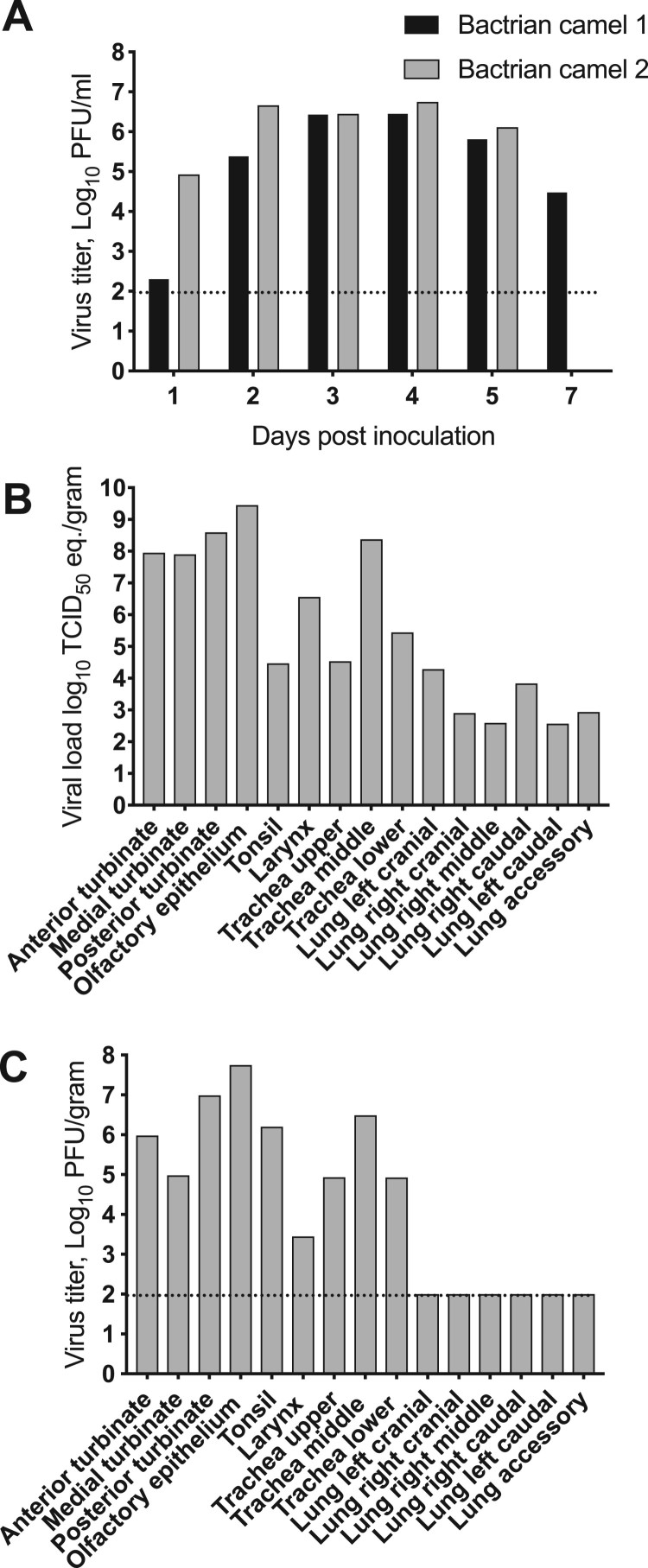
MERS-CoV shedding and tissue distribution in Bactrian camels. (A) Virus shedding from the upper respiratory tract in Bactrian camels inoculated with MERS-CoV determined by plaque assay. (B,C) Replication of MERS-CoV in the upper respiratory tract of Bactrian camels determined by qRT-PCR and plaque assay. The dotted line indicates the detection limits of the assays.

Infectious virus was detected in nasal swab samples beginning on 1 dpi and continued through 5 dpi for both animals. Bactrian camel 2 had detectable infectious virus on 7 dpi, but not on 14, 21, or 28 dpi (Figure 4A). Virus shedding reached its peak at 4 dpi, with 10^6.5^ and 10^6.8^ PFU/ml for Bactrian camel 1 and Bactrian 2 respectively. We compared the shedding of the two Bactrian camels with that of two dromedary camels inoculated in a previous experiment by area under curve analyses (AUC) (3), with Bactrian camels (AUC 23.85, 21.61–26.09 95% CI) and dromedary camels (AUC 23.18, 21.92–24.45 95% CI) being virtually identical.

Tissue distribution was only evaluated in Bactrian camel 2 on 5 dpi. MERS-CoV RNA was detected in primarily in the upper respiratory tract (nasal turbinates, olfactory epithelium, tonsil, larynx and trachea) and limited amounts in the lower respiratory tract (lungs) (Figure 4B). Infectious virus was detected in nasal turbinates, olfactory epithelium, tonsil, larynx and trachea suggesting an upper respiratory tract infection (Figure 4C). No virus replication was observed in any of the other tissues analysed: lungs, heart, spleen, kidney, bladder, duodenum, colon, jejunum or lymph nodes (retropharyngeal, mediastinal, mesenteric, tracheobronchial and prescapular).

### Histopathology and immunohistochemistry

**Figure 5.**
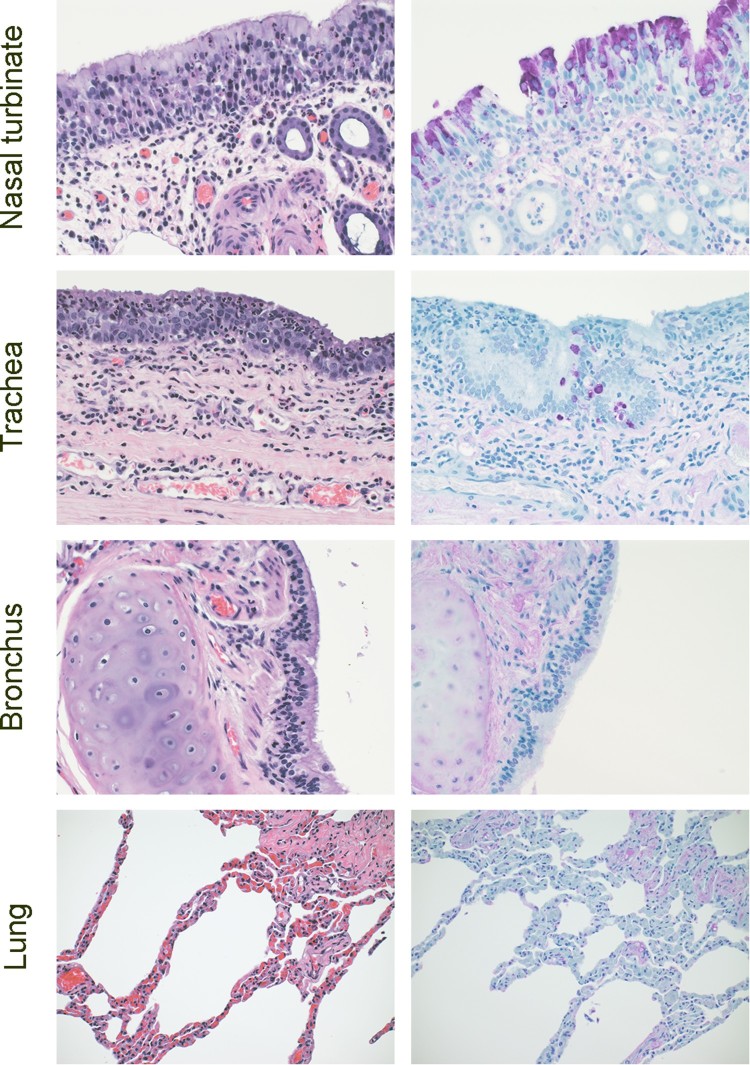
Histopathology in Bactrian camels infected with MERS-CoV. Histopathologic changes at 5 days post inoculation in camel 2 inoculated with MERS-CoV. Tissues were collected and stained with hematoxylin and eosin (left panel). AntiMERS-CoV immunohistochemical results (right panel) are visible as a dark-purple stain. Viral antigen was detected within the epithelial cells of the nasal turbinates (primarily neuroeptithelium) and trachea (columnar epithelium). Original magnification ×400.

Tissues were collected from Bactrian camel 2 on 5 dpi and evaluated for pathology and the presence of viral antigens by immunohistochemistry. The observed lesions in the upper respiratory tract of the camels were characterized as mild to moderate subacute sinusitis, with epithelial necrosis and lympocytic submucosal inflammation in the nasal turbinates and subacute tracheitis with epithelial necrosis and lympocytic submucosal inflammation and squamous metaplasia. Viral antigen was detected within the epithelial cells of the nasal turbinates (primarily neuroeptithelium) and trachea (columnar epithelium) (Figure 5).

### Serology

Both camels were seronegative at the time of infection and only Bactrian camel 1 was monitored for seroconversion as Bactrian camel 2 was euthanized on 5 dpi. Neutralizing antibodies were first detected in sera from Bactrian camel 1 on 14 dpi; titers were 20, 20, and 40 on days 14, 21, and 28, respectively (Table 1).

**Table 1.** Bactrian camel neutralizing antibody titer against MERS-CoV as determined by plaque reduction neutralization test.

Day	Bactrian camel 1	Bactrian camel 2
0	<10	<10
7	<10	NA
14	20	NA
21	20	NA
28	40	NA

## Discussion

Dromedary camels play a prominent role in the circulation, maintenance, and the zoonotic transmission of MERS-CoV [2,3,24]. In the present study we demonstrated that Bactrian camels are also susceptible to infection with MERS-CoV and, like dromedary camels [10,13], shed large quantities of infectious virus in nasal secretions. Moreover, the virus shedding kinetics of MERS-CoV in Bactrian camels was virtually identical to previous experimental studies in dromedary camels. Similar to infections reported in both naturally and experimentally infected dromedaries, infected Bactrian camels displayed only minor clinical disease characterized by mild to moderate quantities of nasal discharge.

The limitation of this study was use of only two animals and of those, only one was monitored for seroconversion. Nonetheless, the susceptibility and the magnitude and pattern of nasal virus shedding was nearly identical in both animals and clearly demonstrated that Bactrian camels are susceptible to MERS-CoV. The susceptibility of the Bactrian camel to MERS-CoV suggests a general pattern of susceptibility in new and old-world camelids. Experimental infections in camelids have resulted in productive infection and shedding of MERS-CoV [10,14–16], in contrast to other livestock species such as goat, sheep, pigs and horses where either no or very limited infection was observed [16,25,26]. Interestingly, the MERS-CoV amounts in the upper respiratory tract and the shedding from the upper respiratory tract in the new world camelids, llama and alpaca, are generally 2 log lower compared to those of the old world camelids, dromedary and Bactrian camel [10,13–16]. In addition, upon experimental MERS-CoV transmission studies with alpaca, although transmission between infected animal and contact animal occurred, the contact animal had 2 log lower virus shedding than the inoculated animals. This potentially suggests a lower ability of MERS-CoV for sustained transmission in this species [15].

Despite the current lack of field evidence of MERS-CoV infection in Bactrian camels, this study demonstrates that Bactrian camels can be readily infected and shed large quantities of virus in nasal secretions. If MERS-CoV were to be introduced into populations of Bactrian camels, we would expect that a potential endemic and sustained pattern of infection may result and they could act as a reservoir, similar to dromedaries, potentially exposing associated human communities to infection.
